# Fine analysis of a genomic region involved in resistance to Mediterranean corn borer

**DOI:** 10.1186/s12870-018-1385-3

**Published:** 2018-08-15

**Authors:** José Cruz Jiménez-Galindo, Rosa Ana Malvar, Ana Butrón, Marlon Caicedo, Bernardo Ordás

**Affiliations:** 1 Misión Biológica de Galicia, Spanish National Research Council (CSIC), Apartado 28, 36080 Pontevedra, Spain; 2 National Institute of Forestry, Agriculture and Livestock Research (INIFAP), Ave. Hidalgo 1213, Cd., 31500 Cuauhtémoc, Chihuahua, Mexico; 3 National Institute of Agricultural Research (INIAP), 170315 Quito, Ecuador

**Keywords:** *Sesamia nonagrioides*, *Zea mays*, Heterogeneous inbred families (HIFs), Near-isogenic lines, Quantitative trait loci, Insect resistance

## Abstract

**Background:**

*Sesamia nonagrioides* Lefebvere (Mediterranean corn borer, MCB) is the main pest of maize in the Mediterranean area. QTL for MCB stalk tunneling and grain yield under high MCB infestation had been located at bin 8.03–8.05 (4–21 cM and 10–30 cM respectively) in a previous analysis of the EP42 x EP39 RILs mapping population. The objective of the present work was to study with higher resolution those QTL, and validating and estimating with higher precision their locations and effects. To achieve this objective, we developed a set of 38 heterogeneous inbred families (HIFs) which were near-homozygous in the genome, except in the region under study. The HIFs were evaluated in multiple environments under artificial infestation with MCB and genotyped with SNPs.

**Results:**

The QTL for grain yield under high infestation was confirmed with higher precision and improved reliability at 112.6–116.9 Mb. On the contrary, the location of the QTL for stalk tunneling was not validated probably due to the fixation of some genomic regions during the development of the HIFs. Our study confirmed that the co-localization of the QTL for stalk tunneling and grain yield in the previous study was due to linked genes, not to pleiotropic effects. So, the QTL for grain yield can be used for improving grain yield without undesirable effect on stalk tunneling.

**Conclusions:**

The HIF analysis is useful for validating QTL and for conducting deeper studies in traits related to corn borer resistance.

## Background

The area planted with maize worldwide exceeds 184.8 million hectares, with a total annual production of 1037.7 million of metric tons in 2014 [1]. Corn borer is the generic name for different species of Lepidoptera that feed on maize producing tunnels on stalks. Corn borers are found in all continents, for example *Ostrinia nubilalis* Hübner (European corn borer, ECB) in America and Europe, *Ostrinia furnacalis* Guenée in Asia, *Sesamia calamistis* Hampson in Africa, etc. Some studies have reported yield losses up to 30% caused by corn borers [2].

ECB is the main corn borer in central Europe while *Sesamia nonagrioides* Lefebvere (Mediterranean corn borer, MCB) is one of the most important pest of maize in Southern Europe, particularly in Spain [3, 4]. ECB and MCB have usually two or more generations per year. The first generation feeds on leaves of young plants, while the larvae of the other generations feed on stem and ears of the plants that have completed (or are closed to complete) their vegetative growth. The second generation produces the main damage and we will focus on the resistance to this generation.

In studies of maize resistance to corn borers the damage and the level of resistance is commonly measured as the length of the tunnels produced by larvae in the stem. The genetic basis of ECB and MCB resistance measured as tunnel length is polygenic [5, 6] and the values of heritability for this trait varied between experiments in a wide range from 0.5 to 0.8 [7–14].

At molecular level, several QTL experiments with RILs have been carried out to detect QTL related to resistance to ECB and MCB. About 10–15 QTL related to ECB resistance were detected per experiment that explained, approximately, 50 and 60% of phenotypic and genotypic variance, respectively [8, 9]. In a QTL experiment with three connected populations and a relatively high number of RILs (521) and markers (2411), the number of QTL related to ECB resistance (10) and the proportion of phenotypic variance explained by the QTL (37%) was still low [11]. The number of QTL related to MCB resistance detected per experiment was low (1–3) and the genotypic variance explained by the QTL was also low (usually between 20 and 30%) [10, 12, 13, 15]. In addition, in several studies QTL detected for tunnel length co-localized with QTL for other agronomic traits such as plant height [12, 13], days to flowering [9, 11] or grain yield [16]. The co-localization can be due to different genes for each trait that are linked or a single gene with pleiotropic effect on both traits. These previous studies did not allow the discrimination between linkage and pleiotropy, although that knowledge is relevant for the potential application of the QTL in breeding: A gen with a pleiotropic and contrary effect on two traits makes impossible the simultaneous improvement of those traits while two linked genes allows it.

The significant QTL detected with standard biparental populations should be verified in additional experiments before to continue with deeper studies of gene discovery and characterization. In biparental mapping populations the effect of multiple segregating QTL can be confounded and this can lead to reduced power of QTL detection or overestimation of the effects [17]. Near-isogenic lines [18] are effective genetic stocks for studying phenotypic effects attributable to a QTL since the genetic background that commonly influences phenotypic assessments of quantitative traits is standardized [19]. Tuinstra and collaborators proposes a quicker method to develop NILs by identifying inbred lines that are highly homozygous, except for a region that segregates for the trait of interest [20]. These types of NILs were called heterogeneous inbred families (HIFs) [20]. The method can be straightforwardly applied to RILs to validate a QTL previously detected in the RILs. HIF analysis has been used to validate QTL related to plant height and yield [21], leaf number [22], number of vascular bundles [23], and kernel traits [24] in maize. The HIF analysis could be particularly useful to validate QTL related to insect resistance because the precision of QTL mapping for traits related to pest resistance is low due to the intrinsic characteristics of the resistance traits which depend both on plant and insect variation. Thus, HIF analyses have been successfully used to validate QTL related to disease resistance, for example, resistance to Northern Leaf Blight [25] and dwarf disease [26] in maize. However, although numerous insect resistance QTL have been mapped in maize with standard biparental populations, no QTL for insect resistance have been verified with NILs or HIF and some authors have pointed out the need for more precise mapping for traits related to insect resistance in maize [27].

In the analysis of a RILs population derived from EP42xEP39 we detected a region spanning from bin 8.03 to 8.05 where a QTL for stalk tunnel length co-localized with a QTL for grain yield under high infestation and a QTL for flowering [15]. The QTL for stalk tunnel length was located between markers umc1984-umc1858 (79–111 Mb), while the QTL for grain yield and flowering were located between umc1858 and bnlg1812 (111–136 Mb) [15]. The objective of this research was to validate and estimate with higher precision the effects of the QTL for stalk tunnel length, the QTL for grain yield under high infestation and the QTL for flowering detected previously in a RIL population [15]. This is achieved by the development and genetic analysis of a set of HIFs, which provide higher mapping precision than RIL mapping populations.

## Results

**Fig. 1:**
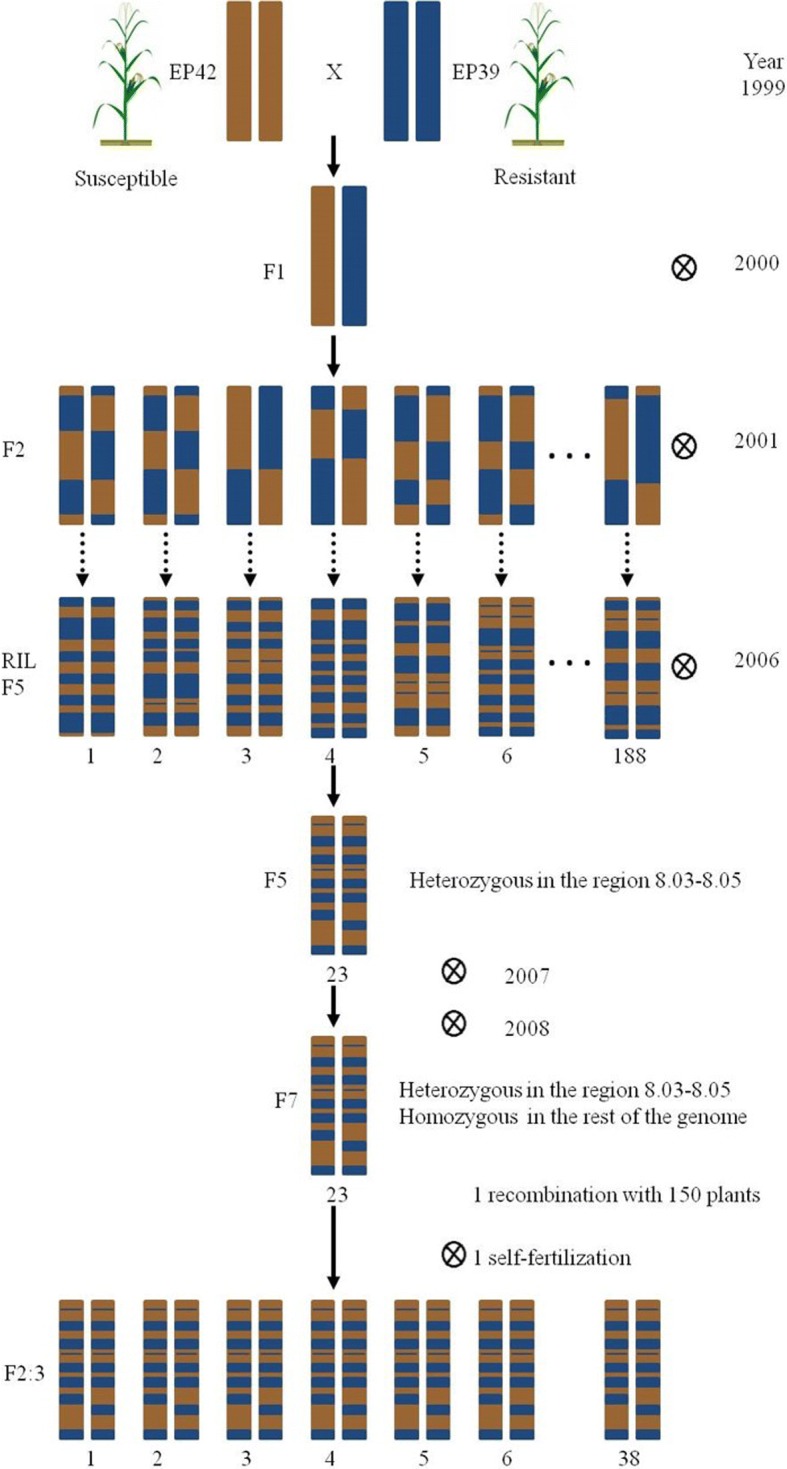
Polymorphic loci for the entire genome of the HIF population, in the chromosome 8 and the genomic region located in bins 8.03–8.05

The genetic analysis of HIFs allows a fine mapping of a specific region previously detected in standard QTL because the genetic background outside the target region is expected to be highly homogenous in the HIFs. We indeed obtained a high level of homogeneity in the genetic background of our set of HIFs which is in contrast with the heterogeneity that the HIFs maintained in the target region where the QTL were located in the previous study (8.03–8.05) (Fig. 1). Thus, the percentage of polymorphic loci ranged from 1 to 4% in all chromosomes except in chromosome 8 which had 19% of polymorphic loci. In the target region where the QTL were located in the previous study the percentage of polymorphic loci was higher: about 50% in bins 8.03 and 8.05 and about 80% in bin 8.04.

As a summary, there was a region from 24 Mb to 139 Mb of chromosome 8 with 84% of polymorphisms, except two smaller sub-regions from 45 Mb to 69 Mb and from 122 Mb to 129 with reduced polymorphisms (10%).

After discarding the SNPs with missing data, there were 73,316 SNPs genotyped in the 38 HIFs. The percentage of polymorphic loci in the whole genome was 0.05%, while the percentage increased to 2% in chromosome 8.

### Linkage mapping

**Fig. 2:**
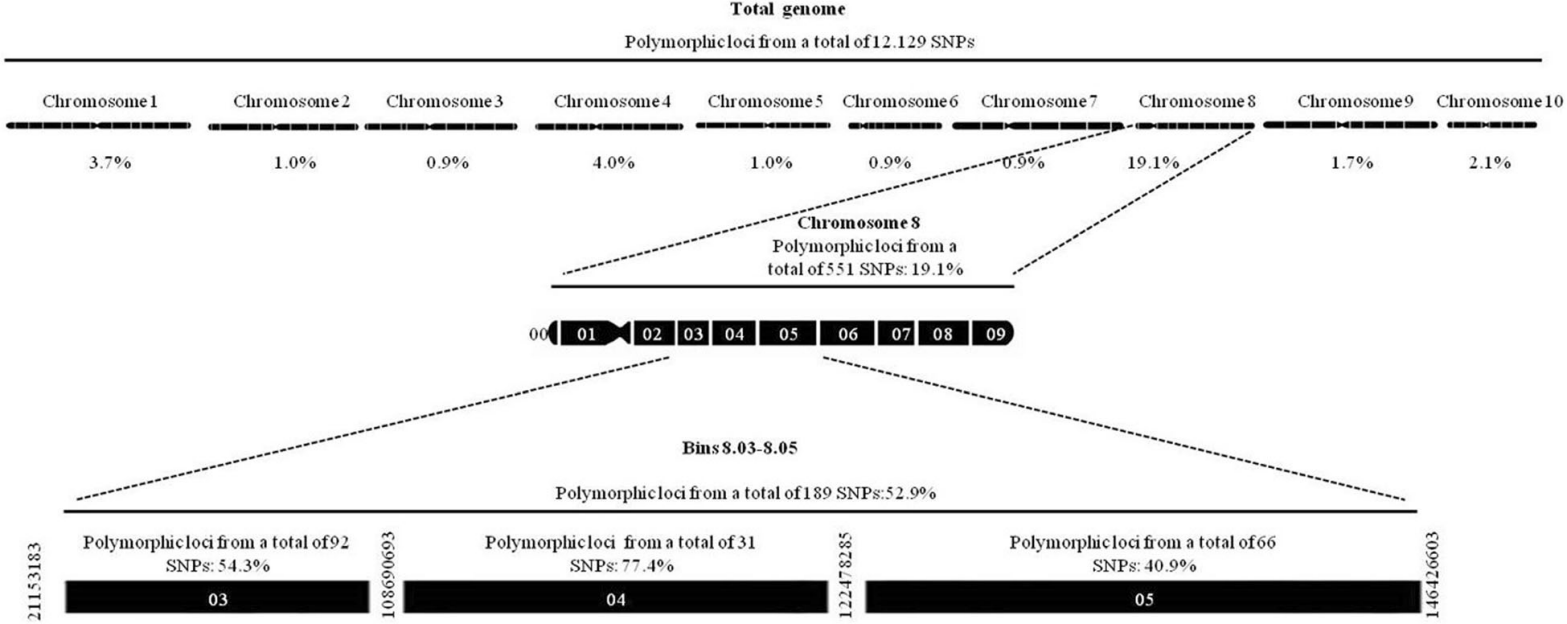
Genetic map of a 38-HIF population derived from the cross EP39 × EP42 where the QTL found for different characters have been located. We used 17 SNP markers at bins 8.03–8.04. The black numbers below the chromosome indicate the position in bp of each SNP marker while the white numbers on the chromosome indicate the bin number. The 95% confidence intervals are indicated by the length of the QTL bar

In the linkage mapping analysis of the HIFs, we found QTL for grain yield, stalk tunneling, and silking in which the allele from EP42 provided more yield, longer galleries and early silking in congruence with the original EP42 x EP39 mapping experiment (Table 1, Fig. 2).

**Table 1:** Summary of QTL mapped in the HIFs derived from EP42xEP39 which were evaluated in a three-year experiment under MCB infestation

QTL position	LOD	Flanking marker’s positions (bp)	Additive mean effect^c^					
bin^a^			DS^b^ ($\widehat{\alpha}$)	ES	TS	Bias	Freq^d^	Phenot. V. (R^2^)^e^
Stalk tunnel length (cm)								
8.03–8.04	1.5	27,637,188–35,814,899	1.6	1.7	0.37	0.79	0.49	11.4
Plant height (cm)								
8.03–8.04	3.0	108,499,269–112,617,651	5.5	5.4	4.7	0.13	0.95	26.7
Silking (days)								
8.04	1.4	112,617,651–116,854,699	−0.44	−0.51	− 0.19	0.63	0.48	10.7
Yield (Mg ha^−1^)								
8.04	3.9	112,617,651–116,854,699	0.31	0.32	0.21	0.23	0.87	34.9

^a^Bin locations were designed by an X.Y code, where X was the linkage group containing the bin and Y was the location of the bin within the linkage group [53]

^b^DS was the estimation for the complete data set; ES was the average value for the 1000 estimation sets; TS was the average value of the 1000 validation sets in cross validation; the bias was calculated as the difference between ES and TS estimations divided by the ES estimation

^c^Additive effect of the QTL estimated as half the difference between the genotypic values of the two homozygotes. A positive estimation means that EP42 carried the allele with higher value

^d^Detection frequency of the QTL in the cross-validation test

^e^Proportion of phenotypic variance explained by each QTL

### Haplotype analysis and identification of causative genes

**Fig. 3:**
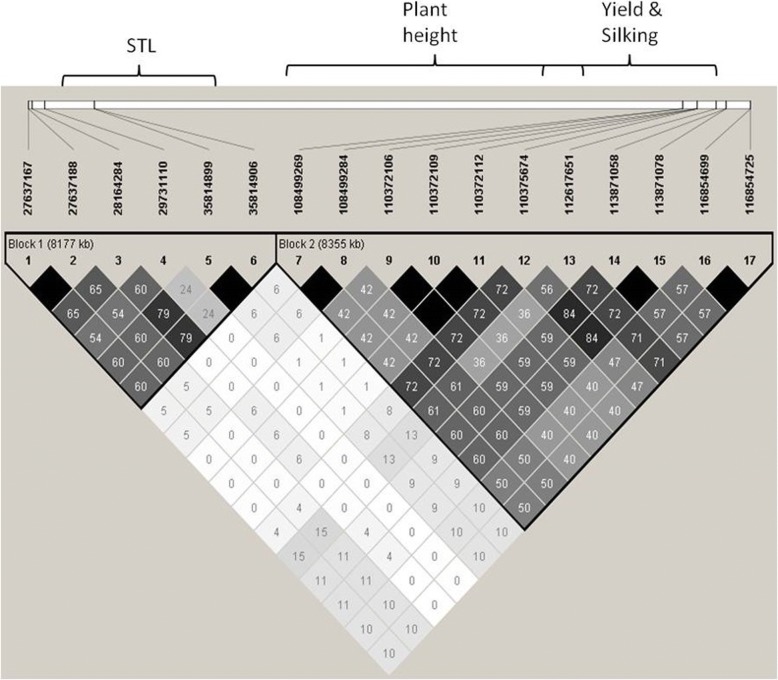
Local linkage disequilibrium in Haploview, measured as r^2^ between pair of SNP and haplotype blocks for a genomic region located at 8.03–8.04 and studied by HIFs analysis. Block in linkage disequilibrium at 50 and 60% of r^2^ [52]

The haplotype analysis showed that there were two haplotype groups in the region under study (Fig. 3). The QTL for grain yield and the QTL for silking were in block 2 overlapping with the QTL for plant height located also in block 2 and the QTL for stalk tunneling was in block 1. Thus, the stalk tunnel and grain yield QTL were in different blocks being possible the recombination between blocks.

The comparison of haplotypes was exclusively made for the grain yield QTL because its location and effect were clearly validated and the homogenization of the genetic background was effective resulting in high proportion of the variance being explained. The yield of the lines with the haplotype of EP42 in the region where the QTL for yield was detected (from 112.6 to 117.7 Mb) did not overlap with the yield of the HIFs with the haplotype of EP39, with the exception of HIF_2 (Table 2). Thus, the mendelization of this QTL was almost achieved with the development of the HIFs families in spite of the moderate effect of the QTL. Two HIFs had recombinants in the region which gives us valuable information. HIF_40 had the haplotype of EP39 except for two SNP at 116.9 Mb where it had the alleles of EP42; also, this HIF had a high yield similar to the HIFs with the haplotype of EP42 in the entire region (from 112.6 to 117.7 Mb). On the contrary, HIF_37 had the haplotype of EP42 except for the two SNP at 116.9 where it was heterozygous; this HIF had a low yield similar to the HIFs with the haplotype of EP39 in the entire region (from 112.6 to 117.7 Mb). Thus, a change in the alleles at 116.9 Mb had a great impact on the yield of HIF_40 and HIF_37 which suggests that the QTL for grain yield under high infestation could be located around this location (113.9–117.7 Mb). In this region 72 genes are located, 33 of them with a function recognized by the PlantRegMap platform (Table 3). Grain yield is the result of multiple processes throughout the life of the plant and potentially any gene could have an effect on this complex trait. Therefore, it is not possible to reduce the number of candidate genes in the region of the QTL based on their known functions. Anyway, the number of candidate genes for the yield QTL has been reduced from thousands in the previous analysis of the biparental population to less than one hundred in the analysis of the HIFs. This relatively reduced number of candidate genes is amenable to differential expression analysis to limit further the number of candidate genes.

**Table 2:**
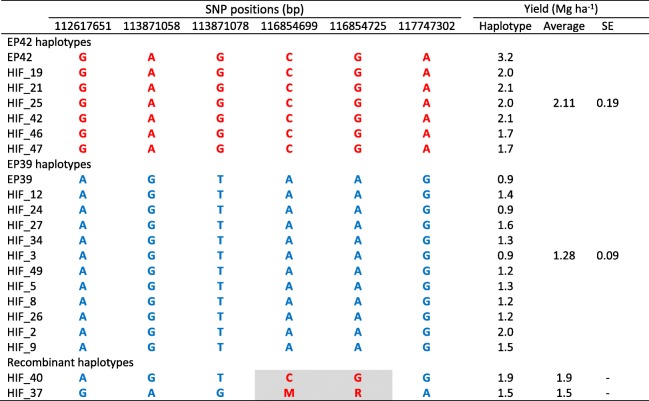
Haplotypes in the region of the QTL detected for yield (Mg ha^− 1^) by interval mapping in a HIF mapping population

Red alleles come from EP42; blue alleles come from EP39. M is heterozygous with A:C. R is heterozygous with A:G

**Table 3:** Candidate genes in the region of the QTL for yield under high infestation with MCB validated in the HIFs

Chromosome	Gene identifier	Map position B73 reference maize genome (v3)	Function
8	GRMZM5G845296 (Mybr60-MYB-related-transciption factor 60)	113,866,855..113869756	glutathione transferase
8	GRMZM2G149286	113,926,527..113943334	cyclin dependent kinase activator. Nuclear localization. Involved in cell cycle regulation and cell differentiation.
8	GRMZM2G149211	113,952,252..113957006	peroxisomal adenine nucleotide transporter involved in fatty acid beta-oxidation during early stage of postgerminative growth
8	GRMZM2G145752	114,114,229..114119298	leucine-rich repeat family protein
8	GRMZM2G452671	114,164,077..114165013	ribosomal protein L34 e superfamily protein
8	GRMZM2G074331	114,244,829..114245802	UDP- Glycosyltransferase superfamily protein
8	GRMZM2G074377	114,267,974..114274299	DNA ligase
8	GRMZM2G402171	114,305,105..114307920	calmodulin-binding family protein
8	GRMZM2G084489	114,352,021 to 114,360,525	CW-type Zinc Finger
8	GRMZM2G134230	114,515,312 to 114,519,184	Succinate dehydrogenase subunit 4
8	GRMZM2G140590	114,541,249 to 114,555,400	Protein kinase
8	GRMZM5G820460	114,654,964 to 114,658,476	F-box domain containing protein expressed
8	GRMZM2G019328	114,701,820 to 114,703,025	Unknown
8	GRMZM2G019596	114,755,993 to 114,763,299	Is a SNARE-like protein that may be involved in vesicular transport to or from the ER (VAP27–2)
8	GRMZM2G091980	114,818,764 to 114,820,923	Unknown
8	GRMZM2G092000	114,824,628 to 114,828,403	Unknown
8	GRMZM2G167689	114,932,399 to 114,933,896	Transporter
8	GRMZM2G111396	115,003,446 to 115,005,892	Encodes one of the BRGs (BOI-related gene) involved in resistance to Botrytis cinerea. (Ara)
8	GRMZM2G413687	115,165,091 to 115,166,099	Unknown
8	GRMZM2G010319	115,297,735 to 115,298,478	Electron transporter
8	GRMZM2G010452	115,313,249 to 115,318,961	ARM repeat superfamily protein
8	GRMZM2G009370	115,377,779 to 115,378,532	GTPase activating protein
8	GRMZM2G363253	115,431,793 to 115,434,496	RING/U-box superfamily protein
8	GRMZM2G174370	115,549,501 to 115,554,290	Tetratricopeptide repeat (TPR)-like superfamily protein
8	GRMZM2G166176	115,645,735 to 115,648,165	glycerol-3-phosphate acyltransferase 5
8	GRMZM2G160763	115,731,220 to 115,735,925	Cytochrome P450 superfamily protein
8	GRMZM2G038401	115,783,521 to 115,792,131	FTSH protease 10
8	GRMZM2G055489	115,832,232 to 115,838,487	Sucrose-6F-phosphate phosphohydrolase family protein
8	GRMZM2G082384	115,948,007 to 115,952,111	ATP binding microtubule motor family protein
8	GRMZM2G382792	115,952,970 to 115,969,607	axi 1 protein
8	GRMZM2G583274	116,015,457 to 116,016,150	Unknown
8	GRMZM2G009936	116,060,353 to 116,062,438	Ribosomal protein L36e family protein
8	GRMZM5G802801	116,145,133 to 116,148,059	heat shock protein
8	GRMZM5G874500	116,148,238 to 116,153,056	cysteinyl-tRNA synthetase
8	GRMZM2G008032	116,198,759 to 116,200,741	Unknown
8	GRMZM2G700614	116,198,948 to 116,199,652	Unknown
8	GRMZM2G007276	116,304,177 to 116,308,901	ubiquitin carrier protein 7
8	GRMZM2G336908	116,376,809 to 116,380,944	Riboflavin biosynthesis protein ribAB
8	GRMZM2G035202	116,382,083 to 116,385,353	Protein-tyrosine phosphatase
8	GRMZM5G883149	116,387,917 to 116,389,990	Nonclathrin coat protein zeta1-COP
8	GRMZM5G886109	116,393,113 to 116,393,881	Unknown
8	GRMZM5G879851	116,507,933 to 116,508,288	Unknown
8	GRMZM2G178815	116,600,180 to 116,602,282	Encodes a member of the MAKR (MEMBRANE-ASSOCIATED KINASE REGULATOR) gene family. MAKRs have putative kinase interacting motifs and membrane localization signals.
8	GRMZM2G178803	116,606,169 to 116,607,231	Late embryogenesis abundant protein
8	GRMZM2G090563	116,676,898 to 116,678,720	Encodes a candidate G-protein Coupled Receptor that is involved in the regulation of root growth by bacterial N-acyl-homoserine lactones (AHLs) and plays a role in mediating interactions between plants and microbes
8	GRMZM2G390400	116,679,405 to 116,697,581	SAC3/GANP/Nin1/mts3/eIF-3 p25 family
8	GRMZM2G090732	116,721,653 to 116,724,613	Protein kinase superfamily protein
8	GRMZM2G175349	116,799,287 to 116,811,743	RING/FYVE/PHD zinc finger superfamily protein
8	GRMZM2G095905	116,824,573 to 116,827,236	Tudor/PWWP/MBT superfamily protein
8	GRMZM2G095921	116,828,824 to 116,832,076	Unknown
8	GRMZM2G422641	116,962,436 to 116,965,331	Kinase interacting (KIP1-like) family protein
8	AC206698.2_FG002	116,970,513 to 116,973,542	Unknown
8	GRMZM2G540732	116,981,901 to 116,982,818	Unknown
8	GRMZM2G051050	117,079,361 to 117,100,135	Ypt/Rab-GAP domain of gyp1p superfamily protein
8	GRMZM5G833625	117,101,465 to 117,101,659	Unknown
8	GRMZM2G163561	117,138,603 to 117,140,359	Ribosomal protein S12/S23 family protein
8	GRMZM2G163658	117,142,617 to 117,160,636	(MCM8) minichromosome maintenance 8. Encodes a minichromosome maintenance protein that is involved with RAD51 in a backup pathway that repairs meiotic double strand breaks without giving meiotic crossovers when the major pathway, which relies on DMC1, fails.
8	GRMZM2G328988	117,273,598 to 117,288,664	(UPL4) ubiquitin-protein ligase 4. Encodes a ubiquitin-protein ligase containing a HECT domain.
8	GRMZM2G064426	117,330,071 to 117,333,930	Encodes a transcription factor from the nuclear factor Y (NF-Y) family, AtNF-YB1. Confers drought tolerance.
8	GRMZM2G063896	117,349,862 to 117,351,374	Histone superfamily protein
8	GRMZM2G068091	117,390,120 to 117,396,833	Haloacid dehalogenase-like hydrolase (HAD) superfamily protein
8	GRMZM2G068192	117,396,878 to 117,401,717	Protein kinase superfamily protein
8	GRMZM2G030673	117,423,561 to 117,428,562	(CPK13) calcium-dependent protein kinase 13
8	GRMZM2G173874	117,498,214 to 117,533,097	(SELT) SELT-like protein precursor
8	GRMZM2G179728	117,535,930 to 117,537,364	GDSL-like Lipase/Acylhydrolase superfamily protein
8	AC197705.4_FG011	117,605,513 to 117,618,076	(UGP3) UDP-glucose pyrophosphorylase 3
8	AC197705.4_FG003	117,630,364 to 117,630,962	PEBP (phosphatidylethanolamine-binding protein) family protein
8	AC197705.4_FG004	117,632,368 to 117,632,889	RING/U-box superfamily protein. Encodes a RING E3 ubiquitin ligase ATL80. Involved in phosphate mobilization and cold stress response in sufficient phosphate growth conditions.
8	AC197705.4_FG001	117,692,828 to 117,694,964	Thiamine pyrophosphate dependent pyruvate decarboxylase family protein
8	AC197705.4_FG006	117,713,897 to 117,715,549	RING/U-box superfamily protein
8	AC197705.4_FG007	117,718,701 to 117,722,383	Outer membrane OMP85 family protein
8	AC197705.4_FG008	117,722,990 to 117,724,339	Arginine N-methyltransferase, putative (DUF688)

## Discussion

New genotyping techniques as GBS allow genotyping with higher density of markers compared to alternative techniques as SSRs. Thus, in the genotyping of the EP42 x EP39 RIL population only 6 SSRs markers were located on chromosome 8 [15], while 17 polymorphic SNPs were genotyped in the target region of chromosome 8 in the HIFs. The highly improved coverture increases the precision of QTL mapping of the present experiment compared to the first experiment.

### Linkage mapping

The position of the QTL for grain yield in the present work was between the markers that flanked the QTL in the EP42 x EP39 RIL mapping population. However, the flanking markers in the HIF analysis delimited a shorter region between 113 and 117 Mb for the grain yield QTL. The additive value estimated in the analysis of the HIF was similar, although slightly higher, to the value estimated in the analysis of the EP42 x EP39 RIL population (0.3 vs 0.2 Mg ha^− 1^). CV was used to validate the estimation of the position and effect of the QTL. The average values of the additive effect estimated from the estimation and test set in the CV were similar to the values estimated by the whole data set (0.21–0.31) which indicates that the estimated values are consistent. Besides, the QTL was detected in 87% of the CV runs, which indicates also that the QTL is reliable. The proportion of CV runs in which the QTL was detected in the EP42 x EP39 RIL population was much lower (40%) indicating that the homogenization of the genetic background in the HIFs was effective for increasing the precision of the QTL detection. The fixation of most of the QTL outside the region target of the analysis in the HIFs also led to an increase in the proportion of phenotypic variance explained by the QTL (from 10.7 to 34.9%). Thus, the isogenization was effective isolating the effect of the QTL spite of its moderate effect and the moderate heritability of grain yield. Huo and collaborators found, after the homogenization of the genetic background, a similar increase in the proportion of phenotypic variance explained by QTL [28], but in a trait of high heritability as kernel number.

The location of a QTL for silking close to the QTL for yield was also confirmed in the analysis of the HIFs. Contrary to the QTL for yield, the reliability and percentage of variance explained by the silking QTL was reduced in the HIF compared to the EP42 x EP39 RIL population. In the EP42 x EP39 RIL population the flowering QTL had a large effect, explaining 30% of the phenotypic variance, in coincidence with other studies which detected a QTL of large effect for silking in the same region [29–33]. This large effect could be due to the combined effect of several flowering genes located near each other as ZCNC8 at 124 Mb [34] and Zm-Rap2.7 at 134 Mb [35]. ZCNC8 is located near of the QTL for flowering detected in the HIFs, but in a region that was unwillingly fixed during the development of the HIFs which could explain the reduced effect detected in the HIFs compared to the RILs.

At difference of the QTL for yield and the QTL for flowering, there were discrepancy in the location of the QTL for stalk tunneling in the analyses of RILs and HIFs. In the analysis of the RILs a QTL for stalk tunneling was located between 79 and 111 Mb, while in the analysis of the HIFs it was located between 28 and 36 Mb. The analysis of the RILs either was not able to detect any effect from 28 to 36 Mb or could locate their effects outside the region due to lack of markers coverage in the region. On the other hand, the analysis of the HIFs could have failed to detect any effect from 79 to 111 Mb due to fixation of genomic regions during the development of the HIFs. There may have been direct fixation of genes related to stalk tunneling or, alternatively, the reduction in the estimated effect of the QTL for flowering could have affected the detection of the QTL for stalk tunneling. Krakowsky and collaborators also failed to detect some QTL for stalk tunneling after adjusting for flowering [9]. These results are consistent with the relationship between time to flowering and stalk damage by corn borers observed at phenotypic [36] and molecular level [8, 13].

We identified a QTL for plant height between 108 and 113 Mb which was not detected in the analysis of the EP42 x EP39 RIL population. This QTL does not seem a false positive because explained almost 30% of the phenotypic variance and the additive effects estimated using whole data, estimation and test sets were similar (5 cm) indicating that the magnitude of the bias in the estimation of the values was not large. Furthermore, the QTL was detected in 95% of the CV runs indicating that the location of the QTL was reliable. Differences between original and validation studies in QTL experiments for disease resistance can be attributed to QTL x environment interaction, high experimental error, overestimation of the effects, and lack of statistical power [37, 38]. Those reasons do not seem to be applicable to our QTL for plant height because the QTL for grain yield was consistently found in the HIFs in spite of the low effect of the QTL in the EP42 x EP39 RIL population and the moderate heritability and large interaction with environment of the trait. Alternatively, the failure to detect the QTL in the EP42 x EP39 RIL population could be due to the presence of two QTL with counteracting effects linkage in repulsion so the combined effect is null [15]. One of them could be fixed in the development of HIFs, allowing the detection of the other one.

### Haplotype analysis and identification of causative genes

Schulz and collaborators have reported significant and negative genetic correlations between tunnel length and grain yield [7] which implies that undesirable reduction in grain yield could accompany the improvement of the resistance. This undesirable, indirect response to selection for resistance, has indeed happened in several selection programs for corn borer resistance [39–42].

At molecular level, some QTL for stalk tunneling were localized in the same regions than QTL for grain yield due to linked genes or genes with pleiotropic and contrary effects in both traits [16] which hampers the use of those QTL in breeding. To know if the co-localization of QTL is due to linked genes or one gene with pleotropic effects is critical for the use of QTL in breeding. If the co-localization of QTL is due to linked genes then the simultaneous improvement of both traits is possible, but it is not if both QTL are due to the same gene with pleiotropic effects. In the analysis of the EP42 x EP39 RILs [15] we found a QTL for yield and a QTL for stalk tunneling in the same region, with the allele that increased yield having a negative effect on resistance. However, in the HIFs we only detected the QTL for yield, but not the QTL for stalk tunneling which is indicative that the gene responsible for the QTL for yield does not have a pleiotropic effect on resistance. Thus, the QTL could be used for improving grain yield without indirect undesirable effects on stalk tunneling.

## Conclusions

The HIF analysis was effective for validating the QTL for grain yield under high infestation which was detected with higher precision and improved reliability. On the other hand, the location of the stalk tunneling QTL was not confirmed probably due to fixation of genes related to stalk tunneling or flowering during the development of HIFs. The HIF analysis allowed the detection of a new QTL for plant height not previously detected, probably due to the confounded effect of multiple segregating QTL. We conclude that the HIF analysis is useful for validating QTL and conducting deeper studies in traits that have associated high experimental error and moderate heritability as those related to corn borer resistance.

## Methods

### Plant materials

**Fig. 4:**
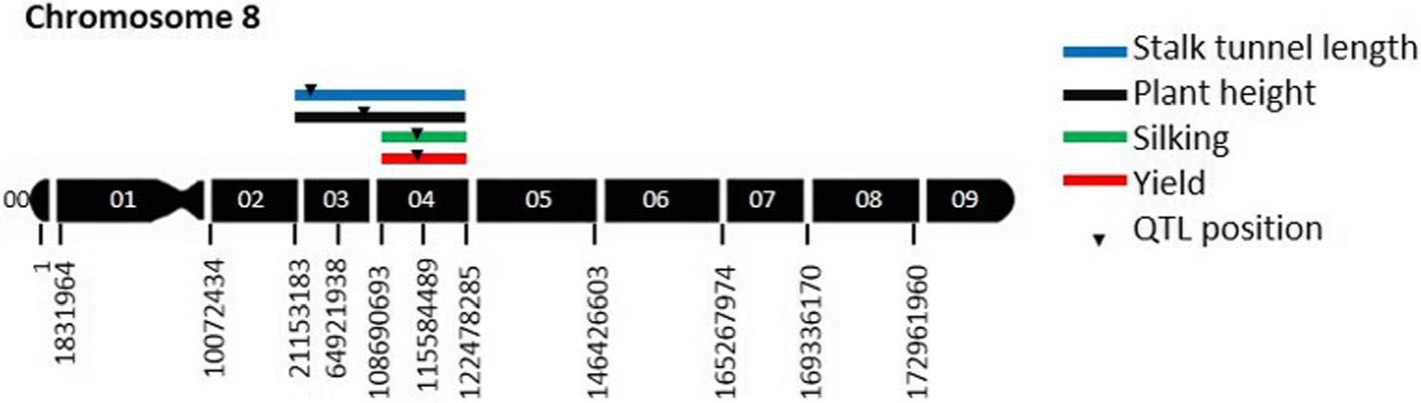
Scheme for developing 38 HIFs from a F_7_ line (LR-23), which was obtained from the cross EP42 × EP39

We used the HIF method for developing the NIL population under study [20]. A RIL heterozygous for three markers (*umc1984*, *umc1858* and *bnlg1812*) located in the region 8.03–8.05 where the QTL for stalk tunneling, grain yield, and flowering were previously detected [15] and, with the highest level of homozygosity everywhere else compared to other families, was selected out of the 188 F_5_ RILs derived from EP39 x EP42. That RIL was named LR-23. The selected family LR-23 was self-pollinated twice to increase the level of homozygosity outside the 8.03–8.05 region. A single F_7_ plant from LR-23, which remained heterozygous in the target region (8.03–8.05), was self-pollinated. Seeds from this plant were sown and crosses among approximately 67 plants were made resulting in 38 HIFs (HIF_1, HIF_2, etc) with enough seed for posterior evaluations. A scheme of the development process of the HIFs is shown in Fig. 4.

### Experimental design

The 38 HIFs were sown at Pontevedra, Spain (42° 24‘N, 8° 38‘W, and 20 m above of sea level) in three different years and cultivated under standard methods.

The 38 HIFs were evaluated along with the parental inbreds EP42, EP39 and LR-23 using a 6 × 7 lattice design with three replications per year. The trials were hand planted and each experimental plot consisted of one row, spaced 0.8 m apart, with 15 two-kernel hills spaced 0.21 m apart. Plots were overplanted and thinned, obtaining a final density of approximately 60,000 plant ha^− 1^. The evaluations were performed under artificial infestation with MCB eggs obtained at the Misión Biológica de Galicia by rearing the insect [43, 44] with some modifications. Before flowering, five plants from each plot were infested with ~ 40 MCB eggs placed between the stem and the sheath of a basal leaf. We collected the following data: days to silking, measured as the days from planting to the day when 50% of plants in the plot showed silks; plant height, measured in five representative plants in the plot as the average length in centimeters from the ground to the top; grain yield, estimated on a plot basis as Mg ha^− 1^ at 140 g H_2_O kg^− 1^; stalk tunnel length, measured as the average length in centimeters of the stem tunnels made by corn borers on the five infested plants.

### Genotyping

The 38 HIFs derived from LR23 and the two parents were genotyped by GBS in Cornell University Institute of Biotechnology. Twenty-two polymorphic SNPs in the region 8.03–8.05 with percentages of missing data lower than 2.5% were used to validate the QTL.

### Statistical analysis

The phenotypic data were analyzed using the mixed model procedure (PROC MIXED) of SAS [45] considering replications and blocks within replications as random effects and families as fixed effects. A best linear unbiased estimator (BLUE) was obtained to estimate each line mean phenotypic value for both individual and combined data.

#### Linkage mapping

As a first approach to validate the QTL we analyzed the HIFs using composite interval mapping with the software PlabMQTL [46] as we did in the analysis of the RILs in the previous study [15]. A LOD threshold of 1.2 was determined by permutation tests that ensures an experiment wise error rate of *p* < 0.30. A five-fold cross validation (CV) approach was employed for obtaining unbiased predictors of the QTL parameters such as additive effect ($\widehat{\alpha}$) [47]. For each trait, CV was performed for the whole data set (DS) of entry BLUE across environments. A total of 30 entries were used as estimation set (ES) for calibration and 8 entries were used as the test set (TS) for validation. One thousand CV runs were performed in order to determine the QTL frequency and shrinkage of estimations for QTL effects of the QTL detected in the original data set [48]. The magnitude of the bias of the estimation of additive effects $\widehat{\alpha}$_*i*_ explained by each individual QTL was calculated as the difference between the average estimates obtained in ES and in TS divided by the estimate in ES.

#### Haplotype analysis and identification of causative genes

Local linkage disequilibrium measured as r^2^ between pair of SNP and common haplotype patterns in the region under study were assessed in Haploview 4.2 [49]. The uniformity of the genetic background of the HIFs allows the direct comparison of haplotypes to map the QTL [50]. Thus, for the QTL that were validated by the linkage mapping analysis we identified the parental haplotypes (EP42 and EP39) and the recombinant haplotypes in the region of the QTL. We compared the phenotypic value of the HIFs with the parental haplotypes and the HIFs with recombinant haplotypes to fine map the QTL. The filtered predicted gene set from the annotated B73 reference maize genome (v3) [51] was used to characterize candidate genes within the validated QTL.

## Abbreviations

BLUE: Best linear unbiased estimator
bp: Base pairs
CV: Cross validation
DS: Data set
ECB: European corn borer
ES: Estimation set
GBS: Genotyping by sequencing
HIF: Heterogeneous inbred families
LOD: Logarithm of odds
MCB: Mediterranean corn borer
NIL: Near isogenic lines
QTL: Quantitative trait loci
RIL: Recombinant inbreed lines
SAS: Statistical analysis system
SNP: Single nucleotide polymorphism
TS: Test set

## References

[1] FAOSTAT. Statistical database. Food and Agriculture Organization of the United Nations, FAO. Available in: http://www.fao.org/faostat/es/#home. Accessed Feb 2017.

[2] Meissle M, Mouron P, Musa T, Bigler F, Pons X, Vasileiadis VP (2010). Pest, pesticide use and alternative options in European maize production: current status and future prospects. J Appl Entomol.

[3] Lopez C, Sans A, Asin L, Eizaguirre M (2001). Phenological model for Sesamia nonagrioides (Lepidoptera : Noctuidae). Environ Entomol.

[4] Velasco P, Revilla P, Monetti L, Butron A, Ordas A, Malvar RA (2007). Corn borers (Lepidoptera : Noctuidae; Crambidae) in Northwestern Spain: population dynamics and distribution. Maydica.

[5] Cartea ME, Malvar RA, Butron A, Vales MI, Ordas A (1999). Inheritance of antibiosis to Sesamia nonagrioides (Lepidoptera : Noctuidae) in maize. J Econ Entomol.

[6] Butrón A, Malvar R, Velasco P, Vales M, Ordás A (1999). Combining abilities for maize stem antibiosis, yield loss, and yield under infestation and non infestation with pink stem borer. Crop Sci.

[7] Schulz B, Kreps R, Klein D, Gumber RK, Melchingeru AE (1997). Genetic variation among European maize inbreds for resistance to the European corn borer and relation to agronomic traits. Plant Breed.

[8] Cardinal AJ, Lee M, Sharopova N, Woodman-Clikeman WL, Long MJ (2001). Genetic mapping and analysis of quantitative trait loci for resistance to stalk tunneling by the European corn borer in maize. Crop Sci.

[9] Krakowsky MD, Lee M, Woodman-Clikeman WL, Long MJ, Sharopova N (2004). QTL mapping of resistance to stalk tunneling by the European corn borer in RILs of maize population B73× De8. Crop Sci.

[10] Ordas B, Malvar RA, Santiago R, Sandoya G, Romay MC, Butron A (2009). Mapping of QTL for resistance to the Mediterranean corn borer attack using the intermated B73 x Mo17 (IBM) population of maize. Theor Appl Genet.

[11] Foiada F, Westermeier P, Kessel B, Ouzunova M, Wimmer V, Mayerhofer W (2015). Improving resistance to the European corn borer: a comprehensive study in elite maize using QTL mapping and genome-wide prediction. Theor Appl Genet.

[12] Samayoa LF, Butron A, Malvar RA (2014). QTL mapping for maize resistance and yield under infestation with Sesamia nonagrioides. Mol Breed.

[13] Samayoa LF, Malvar RA, McMullen MD, Butrón A (2015). Identification of QTL for resistance to Mediterranean corn borer in a maize tropical line to improve temperate germplasm. BMC Plant Biol.

[14] Samayoa LF, Malvar RA, Olukolu BA, Holland JB, Butrón A. Genome-wide association study reveals a set of genes associated with resistance to the Mediterranean corn borer (Sesamia nonagrioides L.) in a maize diversity panel. BMC Plant Biol. 2015;15:–35. 10.1186/s12870-014-0403-3 PMC4340109 25652257

[15] Ordas B, Malvar RA, Santiago R, Butron A (2010). QTL mapping for Mediterranean corn borer resistance in European flint germplasm using recombinant inbred lines. BMC Genomics.

[16] Bohn M, Schulz B, Kreps R, Klein D, Melchinger AE (2000). QTL mapping for resistance against the European corn borer (Ostrinia nubilalis H.) in early maturing European dent germplasm. Theor Appl Genet.

[17] Szalma SJ, Hostert BM, LeDeaux JR, Stuber CW, Holland JB (2007). QTL mapping with near-isogenic lines in maize. Theor Appl Genet.

[18] Perovic D, Stein N, Zhang H, Drescher A, Prasad M, Kota R, Kopahnke D, Graner A (2004). An integrated approach for comparative mapping in rice and barley with special reference to the Rph16 resistance locus. Funct Integr Genomics.

[19] Pumphrey MO, Bernardo R, Anderson JA (2007). Validating the QTL for fusarium head blight resistance in near-isogenic wheat lines developed from breeding populations. Crop Sci.

[20] Tuinstra MR, Ejeta G, Goldsbrough PB (1997). Heterogeneous inbred family (HIF) analysis: a method for developing near-isogenic lines that differ at quantitative trait loci. Theor Appl Genet.

[21] Pea G, Paulstephenraj P, Canè MA, Sardaro MLS, Landi P, Morgante M (2009). Recombinant near-isogenic lines: a resource for the mendelization of heterotic QTL in maize. Mol Gen Genomics.

[22] Li D, Wang X, Zhang X, Chen Q, Xu G, Xu D (2016). The genetic architecture of leaf number and its genetic relationship to flowering time in maize. New Phytol.

[23] Huang C, Chen Q, Xu G, Xu D, Tian J, Tian F (2016). Identification and fine mapping of quantitative trait loci for the number of vascular bundle in maize stem. J Integr Plant Biol.

[24] Raihan MS, Liu J, Huang J, Guo H, Pan Q, Yan J (2016). Multi-environment QTL analysis of grain morphology traits and fine mapping of a kernel-width QTL in Zheng58 × SK maize population. Theor Appl Genet.

[25] Chung C-L, Jamann T, Longfellow J, Nelson R (2010). Characterization and fine-mapping of a resistance locus for northern leaf blight in maize bin 8.06. Theor Appl Genet.

[26] Tao Y, Liu Q, Wang H, Zhang Y, Huang X, Wang B (2013). Identification and fine-mapping of a QTL, qMrdd1, that confers recessive resistance to maize rough dwarf disease. BMC Plant Biol.

[27] Meihls L, Kaur H, Jander G, editors. Natural variation in maize defense against insect herbivores. Cold Spring Harbor symposia on quantitative biology. Laurel Hollow: Cold Spring Harbor Laboratory Press; 2012. 10.1101/sqb.2012.77.014662 23223408

[28] Huo D, Ning Q, Shen X, Liu L, Zhang Z (2016). QTL mapping of kernel number-related traits and validation of one major QTL for ear length in maize. PLoS One.

[29] Vlăduţu C, McLaughlin J, Phillips RL (1999). Fine mapping and characterization of linked quantitative trait loci involved in the transition of the maize apical meristem from vegetative to generative structures. Genetics.

[30] Buckler ES, Holland JB, Bradbury PJ, Acharya CB, Brown PJ, Browne C (2009). The genetic architecture of maize flowering time. Science.

[31] Coles ND, McMullen MD, Balint-Kurti PJ, Pratt RC, Holland JB (2010). Genetic control of photoperiod sensitivity in maize revealed by joint multiple population analysis. Genetics.

[32] Salvi S, Corneti S, Bellotti M, Carraro N, Sanguineti MC, Castelletti S (2011). Genetic dissection of maize phenology using an intraspecific introgression library. BMC Plant Biol.

[33] Dell’Acqua M, Gatti DM, Pea G, Cattonaro F, Coppens F, Magris G (2015). Genetic properties of the MAGIC maize population: a new platform for high definition QTL mapping in Zea mays. Genome Biol.

[34] Bouchet S, Servin B, Bertin P, Madur D, Combes V, Dumas F (2013). Adaptation of maize to temperate climates: mid-density genome-wide association genetics and diversity patterns reveal key genomic regions, with a major contribution of the Vgt2 (ZCN8) locus. PLoS One.

[35] Salvi S, Sponza G, Morgante M, Tomes D, Niu X, Fengler KA (2007). Conserved noncoding genomic sequences associated with a flowering-time quantitative trait locus in maize. Proc Natl Acad Sci U S A.

[36] Ordas B, Alvarez A, Revilla P, Butron A, Malvar RA (2013). Relationship between time to flowering and stalk and ear damage by second generation corn borers. J Econ Entomol.

[37] Mideros SX, Warburton ML, Jamann TM, Windham GL, Williams WP, Nelson RJ (2014). Quantitative trait loci influencing mycotoxin contamination of maize: analysis by linkage mapping, characterization of near-isogenic lines, and meta-analysis. Crop Sci.

[38] Brauner PC, Melchinger AE, Schrag TA, Utz HF, Schipprack W, Kessel B (2017). Low validation rate of quantitative trait loci for Gibberella ear rot resistance in European maize. Theor Appl Genet.

[39] Russell WA, Lawrance GD, Guthrie WD (1979). Effects of recurrent selection for European corn-borer resistance on other agronomic characters in synthetic cultivars of maize. Maydica.

[40] Klenke JR, Russel WA, Guthrie WD (1986). Recurrent selection for resistance to European corn borer in a corn synthetic and correlated effects on agronomic Traits1. Crop Sci.

[41] Sandoya G, Butrón A, Alvarez A, Ordás A, Malvar RA (2008). Direct response of a maize synthetic to recurrent selection for resistance to stem borers. Crop Sci.

[42] Butrón A, Romay MC, Peña-Asín J, Alvarez A, Malvar RA (2012). Genetic relationship between maize resistance to corn borer attack and yield. Crop Sci.

[43] Eizaguirre M, Albajes R (1992). Diapause induction in the stem corn-borer, *Sesamia nonagrioides* (Lepidoptera, Noctuidae). Entomol Gen.

[44] Khan ZR, Saxena RC (1997). Use of a surrogate stem for eliciting ovipositional response of Busseola fusca (Lepidoptera: Noctuidae). J Econ Entomol.

[45] SAS Institute Inc (2016). SAS 9.3 Guide to software updates.

[46] Utz H. PlabMQTL-Software for meta-QTL analysis with composite interval mapping. Version 0.5 s. Institute of Plant Breeding, Seed Science, and Population Genetics, University of Hohenheim. PlabMQTL Manual. 2012.

[47] Utz HF, Melchinger AE, Schön CC (2000). Bias and sampling error of the estimated proportion of genotypic variance explained by quantitative trait loci determined from experimental data in maize using cross validation and validation with independent samples. Genetics.

[48] Melchinger AE, Utz HF, Schön CC (2004). QTL analyses of complex traits with cross validation, bootstrapping and other biometric methods. Euphytica.

[49] Barrett JC, Fry B, Maller J, Daly MJ (2004). Haploview: analysis and visualization of LD and haplotype maps. Bioinformatics.

[50] Paterson AH, DeVerna JW, Lanini B, Tanksley SD (1990). Fine mapping of quantitative trait loci using selected overlapping recombinant chromosomes, in an interspecies cross of tomato. Genetics.

[51] Schnable PS, Ware D, Fulton RS, Stein JC, Wei F, Pasternak S (2009). The B73 maize genome: complexity, diversity, and dynamics. Science.

[52] Gabriel SB, Schaffner SF, Nguyen H, Moore JM, Roy J, Blumenstiel B (2002). The structure of haplotype blocks in the human genome. Science.

[53] Gardiner JM, Coe EH, Melia-Hancock S, Hoisington DA, Chao S (1993). Development of a core RFLP map in maize using an immortalized F2 population. Genetics.

